# Validation of *Mct8/Oatp1c1* dKO mice as a model organism for the Allan-Herndon-Dudley Syndrome

**DOI:** 10.1016/j.molmet.2022.101616

**Published:** 2022-10-18

**Authors:** Gandhari Maity-Kumar, Lisa Ständer, Meri DeAngelis, Sooyeon Lee, Anna Molenaar, Lore Becker, Lillian Garrett, Oana V. Amerie, Sabine M. Hoelter, Wolfgang Wurst, Helmut Fuchs, Annette Feuchtinger, Valerie Gailus-Durner, Cristina Garcia-Caceres, Ahmed E. Othman, Caroline Brockmann, Vanessa I. Schöffling, Katja Beiser, Heiko Krude, Piotr A. Mroz, Susanna Hofmann, Jan Tuckermann, Richard D. DiMarchi, Martin Hrabe de Angelis, Matthias H. Tschöp, Paul T. Pfluger, Timo D. Müller

**Affiliations:** 1 Institute for Diabetes and Obesity, Helmholtz München, Neuherberg, Germany; 2 German Center for Diabetes Research (DZD), Neuherberg, Germany; 3 Division of Metabolic Diseases, Department of Medicine, Technische Universität München, München, Germany; 4 Institute of Experimental Genetics, German Mouse Clinic, Helmholtz München, German Research Center for Environmental Health, Neuherberg, Germany; 5 Institute of Comparative Molecular Endocrinology, University of Ulm, Ulm, Germany; 6 Research Unit NeuroBiology of Diabetes, Helmholtz München, Neuherberg, Germany; 7 Institute of Developmental Genetics, Helmholtz Zentrum München, Neuherberg, Germany; 8 Chair of Developmental Genetics, TUM School of Life Sciences, Technische Universität München, Freising-Weihenstephan, Germany; 9 Deutsches Institut für Neurodegenerative Erkrankungen (DZNE) Site Munich, Feodor-Lynen-Str. 17, 81377 Munich, Germany; 10 Munich Cluster for Systems Neurology (SyNergy), Adolf-Butenandt-Institut, Ludwig-Maximilians-Universität München, Feodor-Lynen-Str. 17, 81377 Munich, Germany; 11 Research Unit Analytical Pathology, Helmholtz München, Neuherberg, Germany; 12 Medizinische Klinik and Poliklinik IV, Klinikum der Universität, Ludwig-Maximilians-Universität München, Munich, Germany; 13 Department of Diagnostic and Interventional Neuroradiology, RWTH Aachen University, 52074 Aachen, Germany; 14 Institute of Experimental Pediatric Endocrinology, Charité - Universitätsmedizin Berlin, Germany; 15 Department of Chemistry, Indiana University, Bloomington, IN, USA; 16 Institute of Diabetes and Regeneration Research, Helmholtz München, Neuherberg, Germany; 17 Chair of Experimental Genetics, TUM School of Life Sciences, Technische Universität München, Freising, Germany; 18 Helmholtz München, München, Germany; 19 Neurobiology of Diabetes, Department of Medicine, Technische Universität München, München, Germany

**Keywords:** Allan-Herndon Dudley Syndrome, Thyroid hormone, *Mct8*, *Oatp1c1*, Energy metabolism, Myelination, Motor coordination

## Abstract

**Objective:**

The Allan-Herndon-Dudley syndrome (AHDS) is a severe disease caused by dysfunctional central thyroid hormone transport due to functional loss of the monocarboxylate transporter 8 (MCT8). In this study, we assessed whether mice with concomitant deletion of the thyroid hormone transporters *Mct8* and the organic anion transporting polypeptide (*Oatp1c1*) represent a valid preclinical model organism for the AHDS.

**Methods:**

We generated and metabolically characterized a new CRISPR/Cas9 generated *Mct8/Oatp1c1* double-knockout (dKO) mouse line for the clinical features observed in patients with AHDS.

**Results:**

We show that *Mct8/*Oatp1c1 dKO mice mimic key hallmarks of the AHDS, including decreased life expectancy, central hypothyroidism, peripheral hyperthyroidism, impaired neuronal myelination, impaired motor abilities and enhanced peripheral thyroid hormone action in the liver, adipose tissue, skeletal muscle and bone.

**Conclusions:**

We conclude that *Mct8/Oatp1c1* dKO mice are a valuable model organism for the preclinical evaluation of drugs designed to treat the AHDS.

## Highlights

• Mct8/Oatp1c1 mice represent a valuable model organism for the Allan-Herndon-Dudley Syndrome.
• Mct8/Oatp1c1 show central hypothyroidism and peripheral hyperthyroidism.
• Mct8/Oatp1c1 show decreased life expectancy.
• Mct8/Oatp1c1 show loss of body weight and fat, enhanced bone catabolism and increased T3 action in liver and adipose tissue.
• Mct8/Oatp1c1 show severe neuronal hypomyelination and motor impairments.

## 1 Introduction

Thyroid hormone T3 (3,3′,5-triiodothyronine) is a nuclear acting hormone with important implications in energy, glucose and lipid metabolism [1]. Derived through deiodination of thyroxine (T4) through the tissue-selective action of the deiodinase enzymes 1 and 2 [2], T3 stimulates cellular growth and development [3], induces adipocyte differentiation [1], and enhances adaptive thermogenesis via beiging/browning of inguinal white adipose tissue (iWAT) [4] or through sensitizing of brown adipose tissue (BAT) to sympathetic nervous system (SNS) outflow [5,6]. In the liver, T3 accelerates de novo glucose production [[7], [8], [9]], increases lipolysis [1] and improves cholesterol metabolism by promoting hepatic cholesterol uptake and clearance [1,10]. Beyond these peripheral effects, T3 acts in the brain to modulate feeding behavior via direct action in the hypothalamus [6,11,12]. While these and other metabolic actions render T3 an attractive pharmacological target for the regulation of systemic energy and lipid metabolism [1,13], adverse T3 effects may include skeletal muscle and bone catabolism, hyperactivity, increased body weight, and cardiovascular complications such as cardiac arrhythmia, tachycardia, and heart hypertrophy [1,13,14].

The Allan-Herndon-Dudley Syndrome (AHDS) is a rare X-chromosome-linked human disease caused by loss-of-function mutations in the monocarboxylate transporter 8 (MCT8) gene [15]. A key function of human MCT8 is to facilitate the transport of T3 and T4 across the blood brain barrier (BBB) [16]. Patients with AHDS accordingly show severely decreased levels of T3 and T4 in the brain with elevated levels of T3, and diminished levels of T4, in the peripheral circulation [16]. Consequential to central hypothyroidism, patients with AHDS display brain maldevelopment, shorter life expectancy, and severe cognitive and locomotor impairment [15]. Unlike patients with AHDS, *Mct8*-deficient mice show diminished T3 entry into the brain, but normal levels of thyroid hormone T4 in both the brain and the periphery, and an inconspicuous phenotype [17]**.** In mice with a concomitant deletion of *Mct8* and the organic anion transporting polypeptide (*Oatp1c1*), the central entry of both T3 and T4 is severely impaired, leading to profound impairments in motor ability and brain development [17]. At current, there is no pharmacotherapy available to treat the AHDS, and development of drugs designed to bypass MCT8 deficiency depend on the availability of a model organism that reliably mimics the human pathophysiology, and that is capable to predict treatment outcome. The aim of this study was to assess whether Mct8/Oatp1c1 dKO mice represent a valuable and reliable animal model for the preclinical assessment of drugs to treat AHDS. We hence report the generation and metabolic characterization of a new, CRISPR/Cas9-generated, global *Mct8/Oatp1c1 dKO* mouse, and confirm that these mice display a variety of key pathological hallmarks of AHDS, including increased mortality, central hypothyroidism, peripheral hyperthyroidism, severe motor impairment, decreased neuronal myelination, and enhanced T3 action in the liver, adipose tissue, and the bone. Our studies support the notion that *Mct8/Oatp1c1 dKO* mice are a valuable model organism for the preclinical evaluation of drugs designed to treat human MCT8 deficiency.

## 2 Methods

### 2.1 Animal studies

**Supplementary Figure S1:**
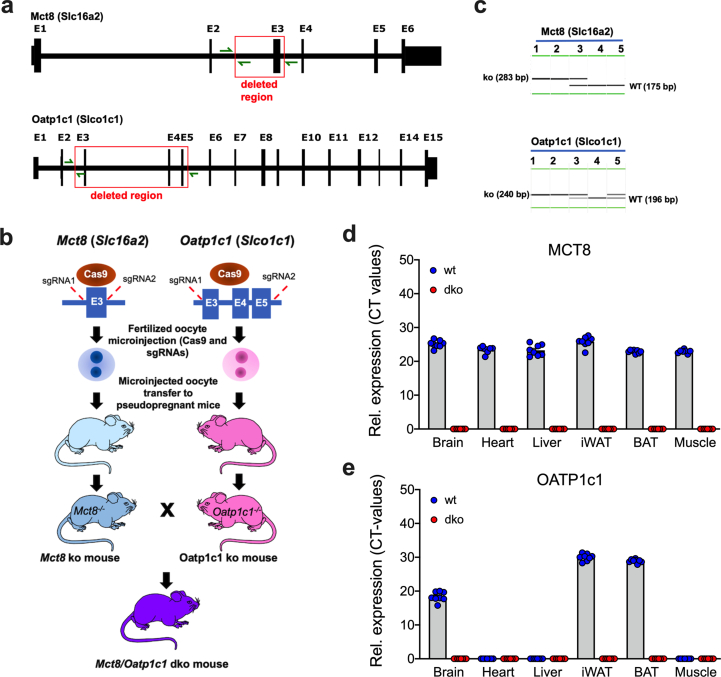
Gene view and targeting strategy for CRISPR/Cas9 gene editing of Slc16a2 (Mct8) and Slco1c1 (Oatp1c1) (a). The CRISPR mediated deleted region is marked with a red square. Green arrows indicate position of genotyping primer. sgRNA pair and Cas9 protein were microinjected into fertilized C57BL/6 oocytes. Microinjected oocytes next were transferred into pseudopregnant female mice and F0 offspring were screened for knockout. Slc16a2 (Mct8) and Slco1c1 (Oatp1c1) single knockout mice were bred to generate Slc16a2; Slco1c1 double knockout (dKO) mouse (b). Representative genotyping result for assessment Mct8/Oatp1c1 ko (c) and expression of target genes Mct8 (d) and Oatp1c1 (e) in key metabolic organs (N = 7–8 mice each group) of 20 wk-old male mice. Undetectable samples were set to zero. Date represent mean ± SEM.

*Mct8/Oatp1c1* dKO mice were generated using CRISPR/Cas9 (Supplementary Figs. 1a and b) and were kept on a C57BL/6 N background. Given that AHDS is an X-chromosome-linked disease that only affects male individuals, only male mice were included in the studies. Mice were double-housed and kept under constant ambient conditions of 22 ± 1 °C, 45–65% humidity and a 12hr/12hr light/dark cycle, with lights on from 6am until 6pm. Mice had free access to water and were fed *ad libitum* with either a standard chow diet (#1314, Altromin GmbH, Lage, Germany) or high-fat diet (D12331, 58% kcal fat; Research Diets, New Brunswick, NJ, USA). For metabolic studies, age-matched mice were grouped based on their genotypes. Body fat and lean tissue mass were measured by nuclear magnetic resonance technology (NMRI; EchoMRI, Houston, TX, USA). For assessment of glucose and insulin tolerance, mice were fasted for 6 h and treated intraperitoneally with either 1.5–2 g of glucose per kg body weight or 0.75 U insulin (Humalog, Eli Lilly, Bad Homburg, Germany) per kg body weight, followed by assessment of blood glucose using standard handheld glucometers. Behavior and motor performance of aged-matched mice were assessed through various tests including open field, grip strength, balance beam, ABR and virtual optokinetic drum. Experiments were performed in accordance to the animal protection law of the European Union and upon permission from the local animal ethics committee of the Government of Upper Bavaria, Germany.

### 2.2 Genotyping

For assessment of genotypes, genomic DNA was isolated from ear-punched dermal samples using the KAPA2 FAST genotyping mix (#KK5121; Sigma-Aldrich, MA, USA). Primers used for genotyping are *Mct8*_forward 5′-GAA-CAG-CTC-AGC-CTT-CCA-AG-3′, *Mct8*_reverse1 5′-TGG-AGT-GGT-TAG-GCA-AGA-GG-3′, *Mct8*_reverse2 5′- CCA-AGT-CCT-CAG-AGC-TCC-AA-3’; *Otap1c1*_forward 5′- GTT-CCT-CCC-AAG-ACC-ACT-CA-3′, *Otap1c1*_reverse1 5′- AGT-CAC-GGT-GCT-CTT-CAG-AT-3′, *Otap1c1*_reverse2 5′- GGC-CTA-TCC-CTG-TAT-GCA-CT-3’. Amplification sizes are 283 bp for *Mct8* ko, 175 bp for *Mct8* wt, 240 bp for *Oatp1c1* ko and 196 bp for *Oatp1c1* wt (Supplementary Fig. 1c).

### 2.3 Indirect calorimetry

Mice were temporarily single-housed in a climate-controlled indirect calorimetric system (Phenomaster, TSE, Bad Homburg, Germany). After 24 h of acclimatization, levels of O_2_ and CO_2_ were measured every 10 min for 5 consecutive days for assessment of energy expenditure, respiratory exchange ratio (RER) and locomotor activity.

### 2.4 Bomb calorimetry

For analysis of assimilation efficiency, food consumption and feces production were measured for 5 consecutive days. Samples were dehydrated at 37 °C for several days, followed by assessment of assimilated energy (kJ/g) and food efficiency (%) [18].

### 2.5 Plasma analysis

Whole blood was collected in EDTA-coated tubes and immediately placed on ice. Plasma was collected upon centrifugation of blood samples at 2000 *g* for 10 min at 4 °C. Plasma insulin levels were measured using the ultrasensitive mouse insulin ELISA kit (#90080, Crystal Chem, Zaandam, Netherlands). Plasma cholesterol and triglycerides were determined using kits from Thermofisher scientific, MA, USA (#10178058) and Wako Pure Chemical Industries, Japan (# 290–63701). For analysis of lipoproteins, plasma samples were pooled and analysed using fast-performance liquid chromatography gel filtration (FPLC) as previously described [19]. Plasma TSH levels were determined using ELISA (#USC-CEA463MU, Cloud-clone corp., TX, USA). According to the manufacturer, the TSH ELISA has high sensitivity and specificity, without cross-reactivity or interference between TSH and analogues.

Plasma NEFA levels were determined by enzymatic assay using reagents from Wako Chemicals, Japan (#917979 and #91898). All assays were performed according to the manufacturer's instruction.

### 2.6 Quantitative analysis of thyroid hormones

For quantification of thyroid hormone levels, tissues were dissected, snap frozen in liquid nitrogen, and stored at-80 °C. For brain analysis, after homogenization of whole-brains at around −200 °C, about 100 mg was used for LC-MS/MS quantification of thyroid hormones (TH) using the Agilent 1290 Infinity II LC system interfaced with an Agilent 6470 triple quadrupole tandem mass spectrometer as described previously [20]. For the analysis of liver, heart, BAT and eWAT a nanoAcquity system interfaced with a QTof2 mass detector was used as previously described [21,22].

### 2.7 Gene expression analysis

Total RNA was isolated from fresh/frozen tissue samples using RNeasy Kit (Qiagen, Hilden, Germany), and cDNA was synthesized using QuantiTect Reverse Transcription Kit (Qiagen, Hilden, Germany). Both the RNA and cDNA concentrations were measured in a NanoDrop™ 2000 Spectrophotometer. The expression of genes was determined by SYBR green based reverse transcription quantitative PCR technique (RT-qPCR) using a Quantstudio 7 flex cycler (Applied biosystems, CA, USA). The following primer sequences were used: *Srebp2_F:* CAT-TCT-CCA-GCA-GTT-CCG-TG, *Srebp2_R:* GCC-CTC-TCA-CAG-TGA-CAG-AA, *Dgat1_F:* CCC-CAT-GCG-TGA-TTA-TTG-CA, *Dgat1_R:* ACA-GGT-TGA-CAT-CCC-GGT-AG, *Ldlr_F:* TCA-GAC-GAA-CAA-GGC-TGT-CC, *Ldlr_R:* CCA-TCT-AGG-CAA-TCT-CGG-TCT-C, *Cyp7a_F:* ATA-CCA-CAA-AGT-CTT-ATG-TCA-CGG, *Cyp7a_R:* CAT-CAC-TTG-GGT-CTA-TGC-TTC-TG, *Ppara_F*: TAC-TGC-CGT-TTT-CAC-AAG-TGC, *Ppara_R:* AGG-TCG-TGT-TCA-CAG-GTA-AGA, *Pgc1a_F*: AGC-CGT-GAC-CAC-TGA-CAA-CGA-G, *Pgc1a_R:* GCT-GCA-TGG-TTC-TGA-GTG-CTA-AG, *Ucp1_F*: GGC-CTC-TAC-GAC-TCA-GTC-CA. *Ucp1_R:* TAA-GCC-GGC-TGA-GAT-CTT-GT, *Cidea_F*: AAT-GGA-CAC-CGG-GTA-GTA-AGT, *Cidea_R*: CAG-CCT-GTA-TAG-GTC-GAA-GGT, *Crh_F*: TCT-GCG-GGA-AGT-CTT-GGA-AAT-GG, *Crh_R*: CAA-GCG-CAA-CAT-TTC-ATT-TCC-CG, *Ghrh_F*: ATG-CTG-CTC-TGG-GTG-CTC-TTT-GTG, *Ghrh_R*: CAT-CTA-CGT-GTC-GCT-GCA-TCC-TG, *Trh_F*: CCC-TGG-ATG-GAG-TCT-GAT-GTC-ACC, *Trh_R:* ACC-CTC-CTC-TCC-CTC-TGT-TTC-TTC-C, *Aldh1l1_F*: CTT-CGC-TGG-CTG-GTG-TGA-TAA-G, *Aldh1l1_R*: AGG-TCA-GGT-TGC-GGT-TGG-G, *Gfap_F*: GAG-AAC-AAC-CTG-GCT-GCG-TAT-AG, *Gfap_R*: TCC-TCC-AGC-GAT-TCA-ACC-TTT-C, *Hprt_F:* AAG-CTT-GCT-GGT-GAA-AAG-GA, *Hprt_R:* TTG-CGC-TCA-TCT-TAG-GCT-TT. Mct8_F: CTC-CTT-CAC-CAG-CTC-CC-TAA-G, Mct8_R: ATG-ACG-AGT-GAT-GGT-TGA-AAG-GC. Oatp1c1_F:GGT-CAT-GGG-CTT-CGG-AAC-TAT-G, Oatp1c1_R: CTC-TCA-TAT-TTC-TCA-TAG-CTG-TAC-TTC-TCC. Hcn4_F: TGT-GTC-ACT-GGG-ATG-GCT-G, Hcn4_R:CCC-AGG-AGT-TAT-TCA-CCA-TGC Kcna1_F: GGT-TAT-TGC-CAT-TGT-GTC-GGT-C, Kcna1_R: AGT-CCT-TGT-CGT-CCT-TCA-GC. Atp2a2_F: GTC-AAG-AAG-CTC-AAG-GAG-AGA-TGG, Atp2a2_R: GTA-AGT-CTT-CAA-ACT-GCT-CAA-TCA-CAA-G. Plb_F: GGC-ATA-ATG-GAA-AAA-GTG-CAA-TAC-CTC, Plb_R:GGT-TCT-GGA-GAT-TCT-GAC-GTG-C. Myh7_F: TGA-GAC-GGA-GAA-TGG-CAA-GAC-G, Myh7_R: ATC-TTG-TCG-AAC-TTG-GGT-GGG. Nppb_F: AAG-GTG-ACA-CAT-ATC-TCA-AGC-TGC, Nppb_R: TTC-CTA-CAA-CAA-CTT-CAG-TGC-GTT-AC.

### 2.8 Micro-computed tomography (μCT) and histomorphometry

Femurs were harvested and analyzed using a μCT device (Skyscan 1176, Bruker, Kontich, Belgium) to assess the bone structure and the bone mineral density (BMD). Images were acquired at 9 μm voxel resolution using a 50 kV X-ray voltage, a 500 μA current, and a 0.5 mm aluminum filter with a 1° rotation step. Following reconstruction using NRecon and DataViewer (Bruker Corporation, Billerica, MA, USA), the trabecular and cortical bone analysis was performed at the 0.7 and 4.8 mm proximal of the growth plate using 2.24 and 0.88 mm regions of interest, respectively. The structural analysis and BMD analysis were performed using the CTAn software (Bruker Corporation, Billerica, MA, USA). Three-dimensional images were created using CTVox (Bruker Corporation, Billerica, MA, USA). For bone histomorphometry, femurs were fixed in 4% PFA, decalcified for 15 days with 20% ethylenediamine tetraacetic acid (EDTA), dehydrated, and embedded in paraffin. Five μm thick bone sections were stained using tartrate resistant acid phosphatase (TRAP) and cellular parameters (Osteoblast and osteoclast numbers and surface per bone surface) were determined using the OsteoMeasure histomorphometry system (OsteoMetrics, Decatur, USA). All the measurements were performed in accordance with the guidelines of the American Society for Bone and Mineral Research (ASBMR) [23].

### 2.9 Ex vivo ultra-high-resolution CT analysis

Ultra-high-resolution CT and MRI were performed ex vivo on 2 dko mice and 2 wildtype mice. CT imaging was performed on an ultra-high-resolution Scanner (Aquilion Precision, Canon Medical Systems, Otawara, Japan) with the following specifications: focal spot 0.4 mm × 0.5 mm, detector elements with 0.25 × 0.25 mm and a beam collimation of 0.25 mm × 160 mm rows, resulting in a slice thickness of 0.25 mm and a special resolution of 150 μm. A Tube voltage of 80 kV and a Tube current of 400 were utilized with a detailed pitch of 0.57 and a rotation time of 0.5 s. MRI was performed on a 3 T MRI Scanner with a Loop Coil (Magnetom Skyra, Siemens Healthineers, Erlangen, Germany) using the following sequence protocol: multiplanar 2D T2-w and T1-w acquisitions of the brain; 3D T1 VIBE and 3D T2 SPACE acquisitions of the body (slice thickness = 0.9 mm).

### 2.10 Histology

Tissue samples were harvested and immediately fixed with 4% (w/v) neutrally buffered formalin (HT501128, Sigma-Aldrich, Germany) and subsequently routinely embedded in paraffin (Tissue Tec VIP.6, Sakura Europe, Netherlands). Sections of 3 μm were stained with hematoxylin and eosin (HE), using a HistoCore SPECTRA ST automated slide stainer (Leica, Germany) with prefabricated staining reagents (Histocore Spectra H&E Stain System S1, Leica, Germany), according to the manufacturer's instructions.

### 2.11 Immunohistochemistry

Mice were transcardially perfused with PBS followed by 4% PFA. Brains were harvested, followed by overnight post-fixation in 4% PFA solution, then transferred into gradient sucrose solutions for cryoprotection, and finally stored in 30% sucrose solution at 4 °C. Brain sections were prepared using a cryostat (Leica CM3050S, Leica Biosystems, Wetzlar, Germany) in the coronal plane according to the Allen mouse brain atlas. Fluoromyelin staining was done using Invitrogen™ FluoroMyelin™ Green Fluorescent Myelin Stain (#F34651; Fisher Scientific GmbH, Schwerte, Germany) according to the manufacturer's protocol. Brain sections were shortly rinsed with PBS, incubated in blocking buffer according to the primary antibody type for 1hr at room temperature, followed by incubation with primary antibodies (anti-Myelin Basic Protein Antibody 0.25 μg/ml; anti-Calbindin-D-28 K antibody 1:1000; anti-TRH antibody 1:500; anti-GAD67 antibody 1:2000; anti-Parvalbumin antibody 1:1000) for overnight at 4 °C. Next, the sections were serially rinsed in PBS and incubated with the respective Alexa fluor secondary antibodies (1:500, Thermofisher Scientific, MA, USA). Sections were rinsed serially in PBS, incubated with DAPI (1:10,000, Thermofisher Scientific, MA, USA) for nuclear staining, washed in PBS, mounted with fluorescent mounting medium (Agilent, Ca, USA) and stored at 4 °C. Microscopy images were acquired using a fluorescence microscope (Keyence BZ-9000, Neu-Isenburg, Germany) and a confocal microscope (Leica TCS SP8 SMD, Leica Microsystems CMS GmbH, Manheim, Germany).

### 2.12 Assessment of behavioral and motoric ability

The open field test was carried out according to the standardized phenotyping screens utilised by the International Mouse Phenotyping Consortium (IMPC). For open field tests, parameters recorded include distance traveled, resting and permanence time as well as speed of movement for the whole arena, the periphery and the center using the Actimot system (TSE Systems). The grip strength meter exploits the tendency of a mouse to grasp a horizontal metal grid while being pulled by its tail. During the trial set-up, the mouse grasped a special adjustable grid mounted on a force sensor. The mouse was allowed to catch the grid with either 2 or 4 paws. Three trials were undertaken for each mouse and measurement within 1 min. The mean values were used to represent the grip strength of a mouse. The rotarod device (Bioseb, Chaville, France) is equipped with a computer-controlled motor-driven rotating rod. Mice were placed on the Rotarod at an accelerating speed from 4 to 40 rpm for 300 s with 15 min between each trial. In motor coordination testing, mice were given three trials at the accelerating speed at one day. The mean latency to fall off the Rotarod during the trials was recorded and used in subsequent analysis. The threshold, amplitude, and latency of auditory brainstem response (ABR) were determined using an acoustic chamber and workstation (IAC Acoustics, Niederkrüchten, Germany). Mice were anaesthetised with ketamine/xylazine and were transferred onto a heating blanket in the acoustic chamber and three subdermal needle electrodes were placed on the scalp. For threshold determination, the clicks (0.01 ms duration) or tone pips (6, 12, 18, 24, and 30 kHz of 5 ms duration, 1 ms rise/fall time) stimuli over a range of intensity levels from 5 to 85 dB SPL in 5 dB steps produced by Tucker Davis Technologies hardware with a customized software provided by Welcome Trust Sanger Institute, were used. The sound intensity threshold is chosen manually from the first appearance of the characteristic waveform. Vision tests are performed between 9 am and 4 pm using a virtual optomotor system (Striatech, Tübingen, Germany) as described previously [24]. In the beam walk test (also known as the raised-beam test), mice were trained to traverse a distance of 90 cm on series of elevated, narrow beams (diameters beams 1–4: square 20 mm, round 22 mm, square 12 mm, round 15 mm) to reach an enclosed escape platform. Traversing time as well as foot slips and falls were recorded. Each mouse performed three consecutive trials, and the average time of these three trials was calculated. All experimental equipment was thoroughly cleaned with Pursept-A and dried prior subsequent tests.

### 2.13 Statistics and software

Statistical analyses were performed using GraphPad Prism version 9 and SPSS version 28.0.1.1. The exact tests used are specified in the figure legends. Analysis of body lengths and energy expenditure were performed using ANCOVA with body weight as covariate as previously suggested [25,26]. Genomic data was visualized using Integrative Genomics viewer (IGV). ImageJ, AutoQuantX3, and Definiens Tissue Studio were used to analyze the digital microscopic images. CT and MRI data were analyzed on a PACS Viewer (SECTRA Workstation IDS7; Sectra AB, Linköping, Sweden).

## 3 Results

### 3.1 *Mct8/Oatp1c1* dKO mice are lean and protected from diet-induced obesity

**Figure 1:**
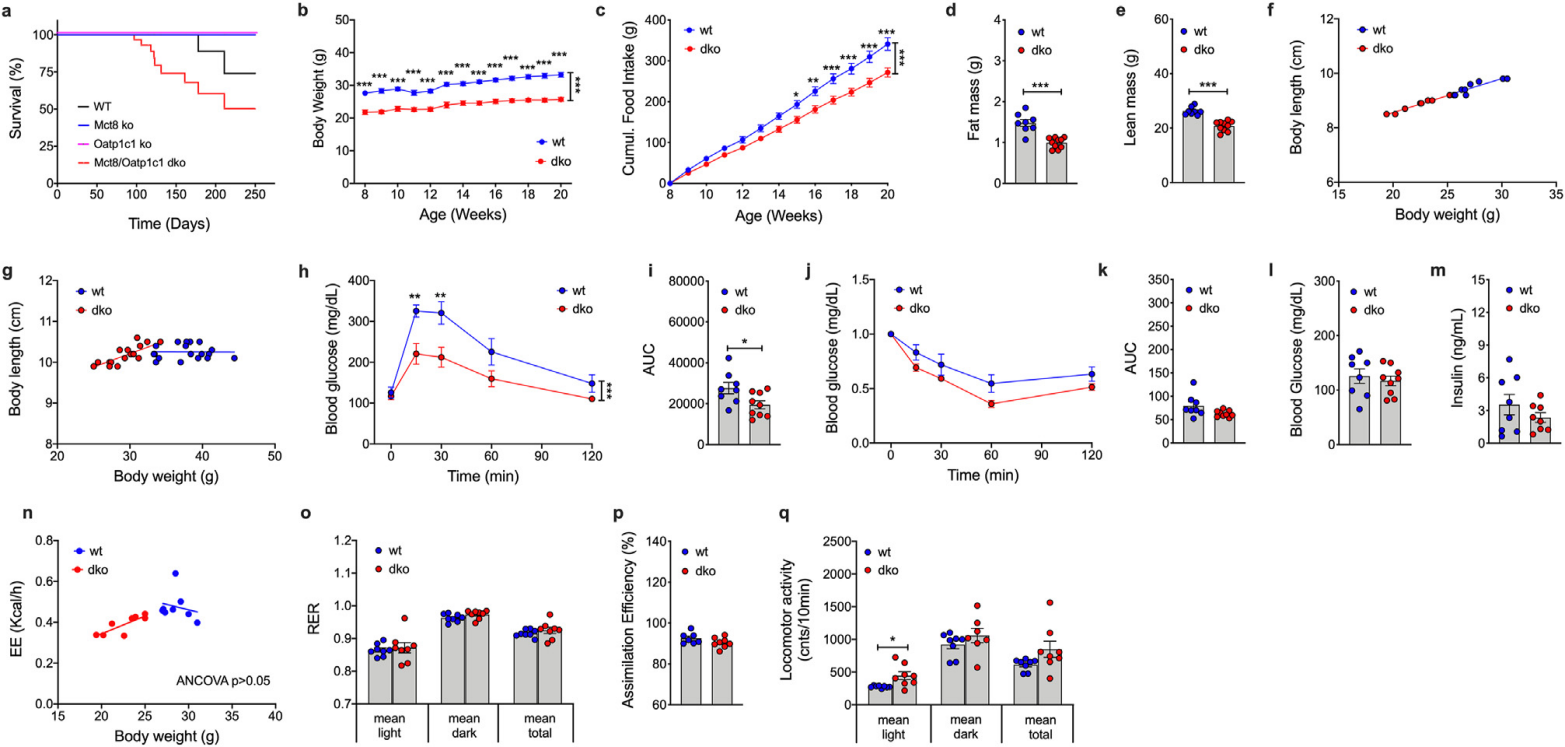
**Metabolic characterization of chow-fed *Mct8/Oatp1c1* dKO mice.** Survival over time of chow-fed C57BL/6 male *Mct8/Oatp1c1* dKO mice relative to wildtype controls and to mice deficient for only either *Mct8* or *Oatp1c1* (n = 30 each group) **(a)**. Body weight **(b)**, cumulative food intake **(c)** as well as body fat and lean tissue mass at the age of 8 wks **(d, e)** in male *Mct8/Oatp1c1* dKO mice and wildtype controls (n = 7–10 each group). Food intake **(c)** was assessed per cage in n = 4/5 cages containing n = 8/10 mice. Body lengths vs. body weight in male *Mct8/Oatp1c1* dKO mice at the age of 8-wks (n = 8–10 each group) **(f)** and 24-wks (n = 10–18 each group) **(g)**. Intraperitoneal glucose tolerance in 12-wk old male mice **(h, i)**, Intraperitoneal insulin tolerance in 14-wk old male mice **(j, k)**, as well as fasting levels of blood glucose **(l)** and insulin **(m)** in 12-wk old male mice (n = 8–9 each group). Energy expenditure **(n)**, respiratory exchange ratio (RER) **(o)**, assimilation efficiency **(p)** and locomotor activity **(q)** in 10-wk old male mice (n = 7–9 each group). Data in panel **b,c,h,j** were analyzed by 2-way ANOVA with Bonferroni's post-hoc test for comparison of individual time points. Data in panel **d,e,i-k,m,o-q** were analyzed by student's two-tailed, two-sided ttest. Data in panel **f,g,n** were analyzed using ANCOVA with body weight as covariate. Date represent mean ± SEM; asterisks indicate ∗ p < 0.05; ∗∗p < 0.01 and ∗∗∗p < 0.001.

**Supplementary Figure S2:**
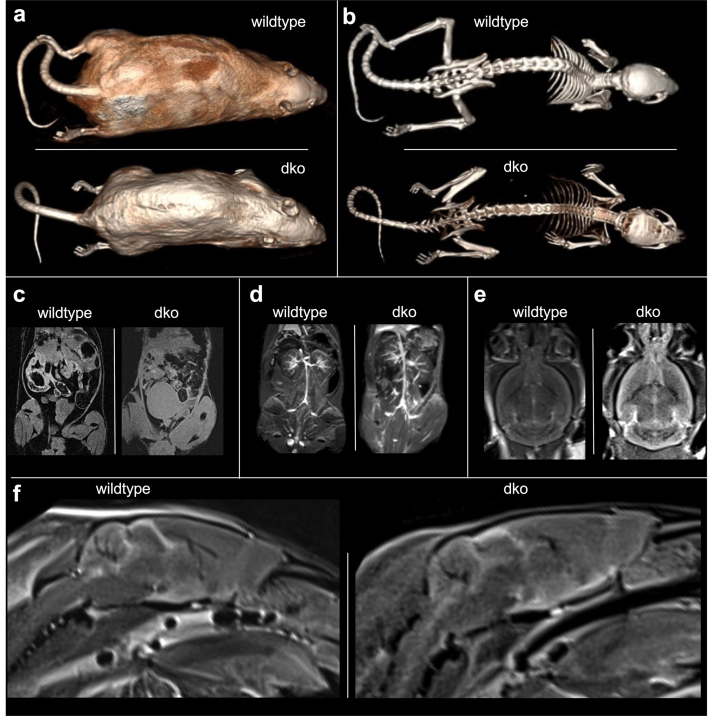
Representative ultra-high-resolution CT and 3T MRI imaging results of a 32 wk-old male dKO mouse and corresponding wildtype control (N = 2 each genotype). Volume rendering reconstruction of the CT data **(a and b)**. Body MRI using a coronal 3D T1-w sequence with spectral fat suppression, T1 VIBE FS **(c)** and a coronal 3D T2-w sequence, T2 SPACE **(d)**. Axial and coronal T2-w MRI of the brain **(e and f)**.

C57BL/6 N mice with concomitant global deletion of *Mct8* (Slc16a2; ENSMUSG00000033965) and *Oatp1c1* (Slco1c1, ENSMUSG00000030235) were generated by Cyagen Bioscience Inc. using CRISPR/Cas9-mediated genome engineering to delete *Mct8* coding exon 3 and *Oatp1c1* coding exons 3–5 (Suppl. Fig. S1A). Upon confirmation of successful target gene deletion using long-range PCR and Sanger sequencing, F1 mice were crossed to obtain *Mct8/Oatp1c1* dKO mice (Suppl. Fig. S1B). Successful gene knock-out (Suppl. Fig. S1C) was verified using qPCR in key metabolic organs, including brain, heart, liver, iWAT, BAT and muscle (Suppl. Figure 1D–E). Consistent with the physiological traits observed in patients with AHDS, we see life expectancy of *Mct8/Oatp1c1* dKO mice decreased relative to wildtype controls, and also relative to mice deficient for either *Mct8* or *Oatp1c1* alone (Figure 1A). When fed with a regular chow diet, *Mct8/Oatp1c1* dKO mice show decreased body weight (Figure 1B) and food intake (Figure 1C) relative to wildtype controls, which is associated with decreased fat and lean tissue mass (Figure 1D,E) without alterations in body lengths (Figure 1F,G). Consistent with the lower body weight, *Mct8/Oatp1c1* dKO mice show improved glucose tolerance (Figure 1H,I). Insulin sensitivity (Figure 1J,K) and the levels of fasting blood glucose and insulin remained unaltered (Figure 1L,M). Similarly, no differences are observed in energy expenditure (Figure 1n), nutrient utilization (Figure 1o) assimilation efficiency (Figure 1p), or total locomotor activity (Figure 1q). Ex vivo ultra-high-resolution CT (Suppl. Figure 2A, B) and whole-body MRI (Suppl. Figure 2c, d) analyses revealed no obvious organ or skeletal abnormalities. However, on CT and MRI, subcutaneous and visceral adipose tissue was markedly reduced in dKO mice increased in wildtype mice.

**Figure 2:**
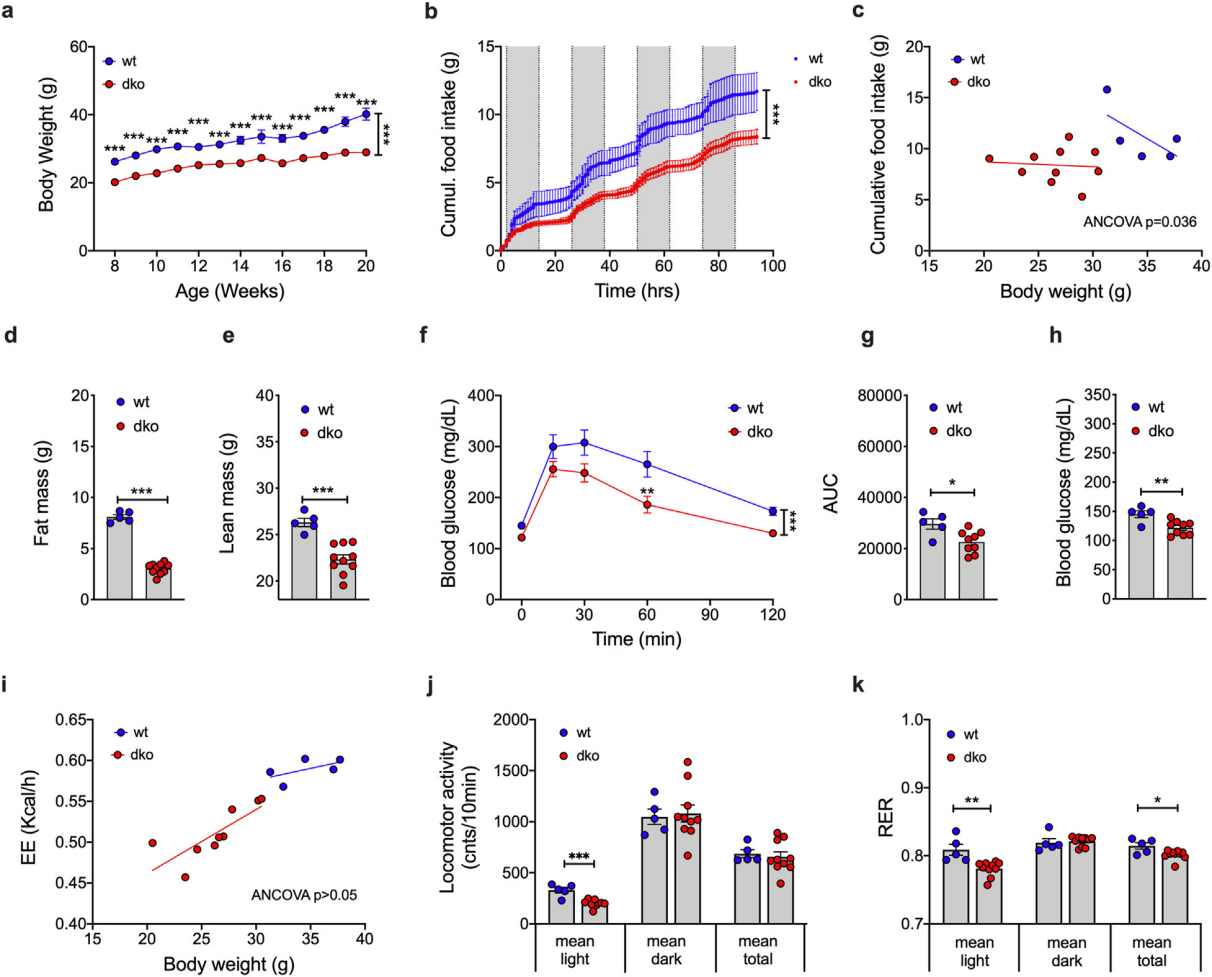
**Metabolic characterization of HFD-fed *Mct8/Oatp1c1* dKO mice**. Body weight **(a)**, food intake assessed cumulativey **(b)** and by ANCOVA with body weight as co-variate **(c)**, as well as body fat and lean tissue mass at the age of 8 wks **(d, e)** in male C57BL/6 *Mct8/Oatp1c1* dKO mice and wildtype controls (n = 5–10 each group). Intraperitoneal glucose tolerance in 12-wk old male mice **(f, g)**, as well as fasting levels of blood glucose **(h)** in 12-wk old male mice (n = 5–10 each group). Energy expenditure **(i)**, locomotor activity **(j)**, respiratory exchange ratio **(k)** in 10-wk old male mice (n = 5–10 each group). Data in panel **a,b, and f** were analyzed by 2-way ANOVA with Bonferroni's post-hoc test for comparison of individual time points. Data in panel **d,e,g,h,j and k** were analyzed using student's two-tailed, two-sided ttest. Data in panel **c** and **i** were analyzed using ANCOVA with body weight as covariate. Date represent mean ± SEM; asterisks indicate ∗ p < 0.05; ∗∗p < 0.01 and ∗∗∗p < 0.001.

When exposed to a high-fat diet (HFD), *Mct8/Oatp1c1* dKO mice also gain less body weight compared to wildtype controls (Figure 2A), which is paralleled by lower food intake (Figure 2B,C), decreased fat and lean tissue mass (Figure 2D,E), improved glucose tolerance (Figure 2F,G) and decreased fasting levels of blood glucose (Figure 2H). No differences are observed in energy expenditure (Figure 2I) and total locomotor activity (Figure 2J), but respiratory exchange ratio is decreased in the HFD-fed *Mct8/Oatp1c1* dKO mice (Figure 2K), indicating enhanced lipid utilization.

### 3.2 Mct8/Oatp1c1 dKO mice show enhanced T3 action in key peripheral metabolic organs

**Figure 3:**
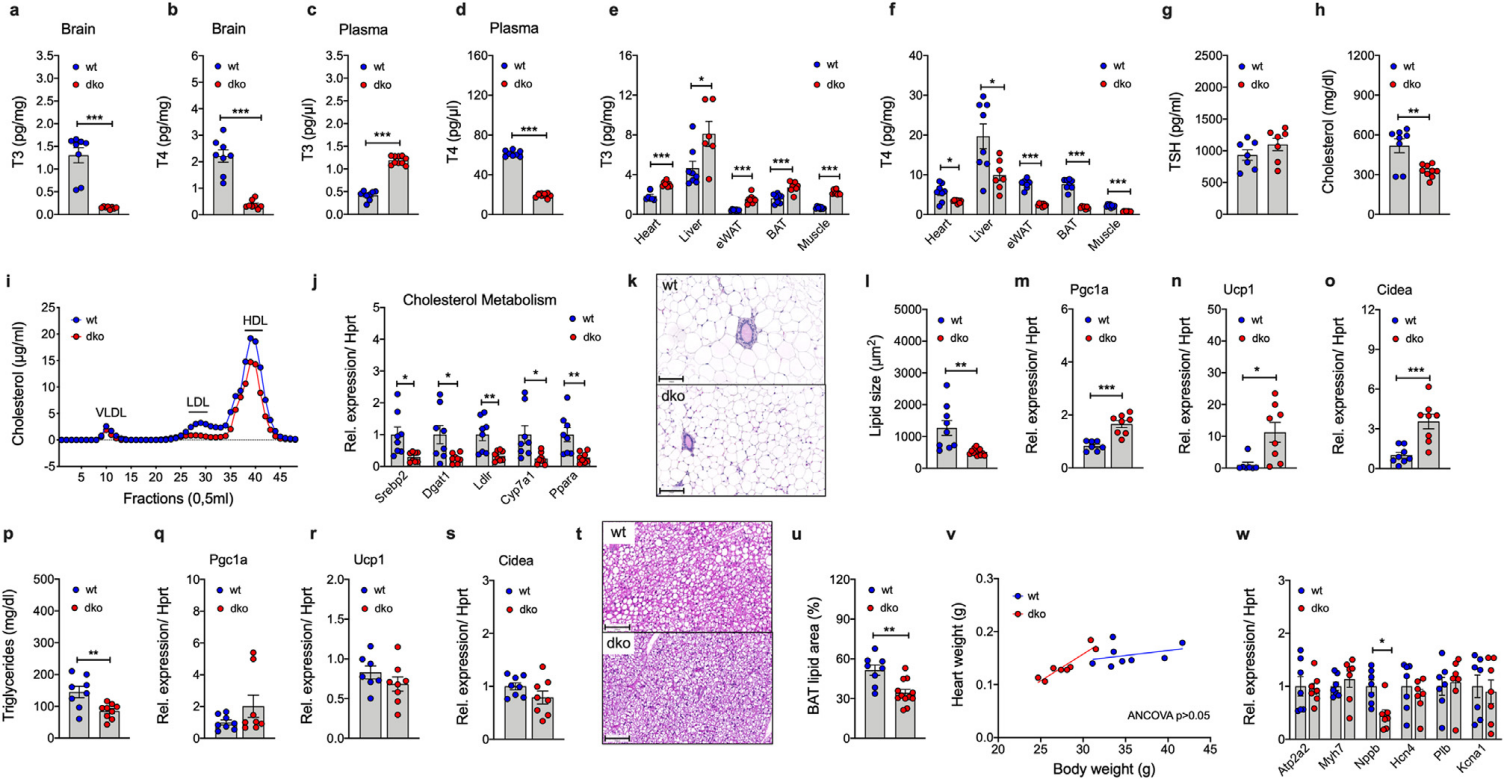
**Quantification, and metabolic action, of T3 and T4 in the brain and periphery.** LC/MS quantification of T3 and T4 in brain **(a,b),** plasma **(c,d)** and peripheral tissues **(e,f)** of 20-wk old male C57BL6 *Mct8/Oatp1c1* dKO mice and wildtype controls (n = 7–10 each group). Plasma levels of TSH and cholesterol in 20-wk old male mice (n = 7–9 each group) **(g,h)** and FPLC analysis of lipoprotein fractions in pooled samples from 20-wk old male mice (n = 8–9 mice pooled from each genotype) **(i)**. mRNA quantification of marker indicative of cholesterol metabolism in liver samples harvested from 20-wk old male mice (n = 8 each group) **(j)**. Representative histological H&E staining of iWAT **(k)** and corresponding quantification of iWAT lipid size **(l)** in 20-wk old male mice (n = 9–13 each group). Expression of genes indicative of browning/beiging in iWAT of 20-wk old male mice (n = 7–8 each group) **(m**–**o)** and plasma levels of triglycerides in 20-wk old male mice (n = 8–9 each group) **(p)**. Expression of genes indicative of thermogenesis in BAT of 20-wk old male mice (n = 7–8 each group) **(q**–**s).** Representative H&E staining of BAT **(t)** and corresponding quantification of BAT lipid content **(u)** in 20-wk old male mice (n = 8–12 each group). Heart weight against body weight in 20-wk old male mice (N = 8 mice each genotype) **(v)** and expression of T3-regulated marker genes implicated in heart hypertrophy in 20-wk old male mice (N = 7 mice each genotype) **(w)**. Data in panel **a-h,j,l-s, u and w** were analyzed using student's two-tailed, two-sided ttest. Data in **v** were analyzed using ANCOVA with body weight as co-variate. Date represent mean ± SEM; asterisks indicate ∗ p < 0.05; ∗∗p < 0.01 and ∗∗∗p < 0.001.

Key hallmarks of the AHDS are pathologically decreased levels of T3 and T4 in the brain, and elevated (T3) and decreased (T4) thyroid hormone levels in the periphery [15]. Consistent with this, we find levels of T3 and T4 decreased in whole-brain tissue of *Mct8/Oatp1c1* dKO mice (Figure 3A,B), along with elevated levels of plasma T3, and decreased levels of plasma T4 (Figure 3C,D). Increased levels of T3 and decreased levels of T4 are also observed in peripheral tissues, namely the heart, liver, white and brown adipose tissue (BAT) and skeletal muscle (Figure 3E,F). No difference is observed in plasma levels of thyroid stimulating hormone (TSH) (Figure 3G). Consistent with the increased levels of hepatic T3, we see total cholesterol, and in particular LDL cholesterol, decreased in *Mct8/Oatp1c1* dKO mice (Figure 3H,I). Nonetheless, we see markers indicative of cholesterol biosynthesis (*Srebp2*), transport (*Dgat1, Ldlr*) and excretion (*Cyp7a1, Ppara*) decreased in the livers of *Mct8/Oatp1c1* dKO mice (Figure 3J), potentially indicating activation of counterregulatory mechanisms to mitigate hepatic cholesterol clearance. An increase in T3 action is also observed in the inguinal white adipose tissue (iWAT), in which the elevated levels of T3 are paralleled by a decrease in lipid size (Figure 3K and l) and enhanced expression of markers indicative of beiging/browning (*Pgc1a, Ucp1, Cidea*) (Figure 3m–o). This is further paralleled by a decrease in plasma triglycerides (Figure 3p). Consistent with the observation that energy expenditure is unaltered in *Mct8/Oatp1c1* dKO mice (Figure 1n), we see no changes in expression of *Pgc1a*, *Ucp1*, and *Cidea* in BAT (Figure 3q–s). But in line with the decreased fat mass (Figure 1D), we find BAT lipid volume decreased (Figure 3t and u). No major differences are observed in heart weight (Figure 3v) or in expression of marker indicative of heart hypertrophy (Figure 3w).

**Figure 4:**
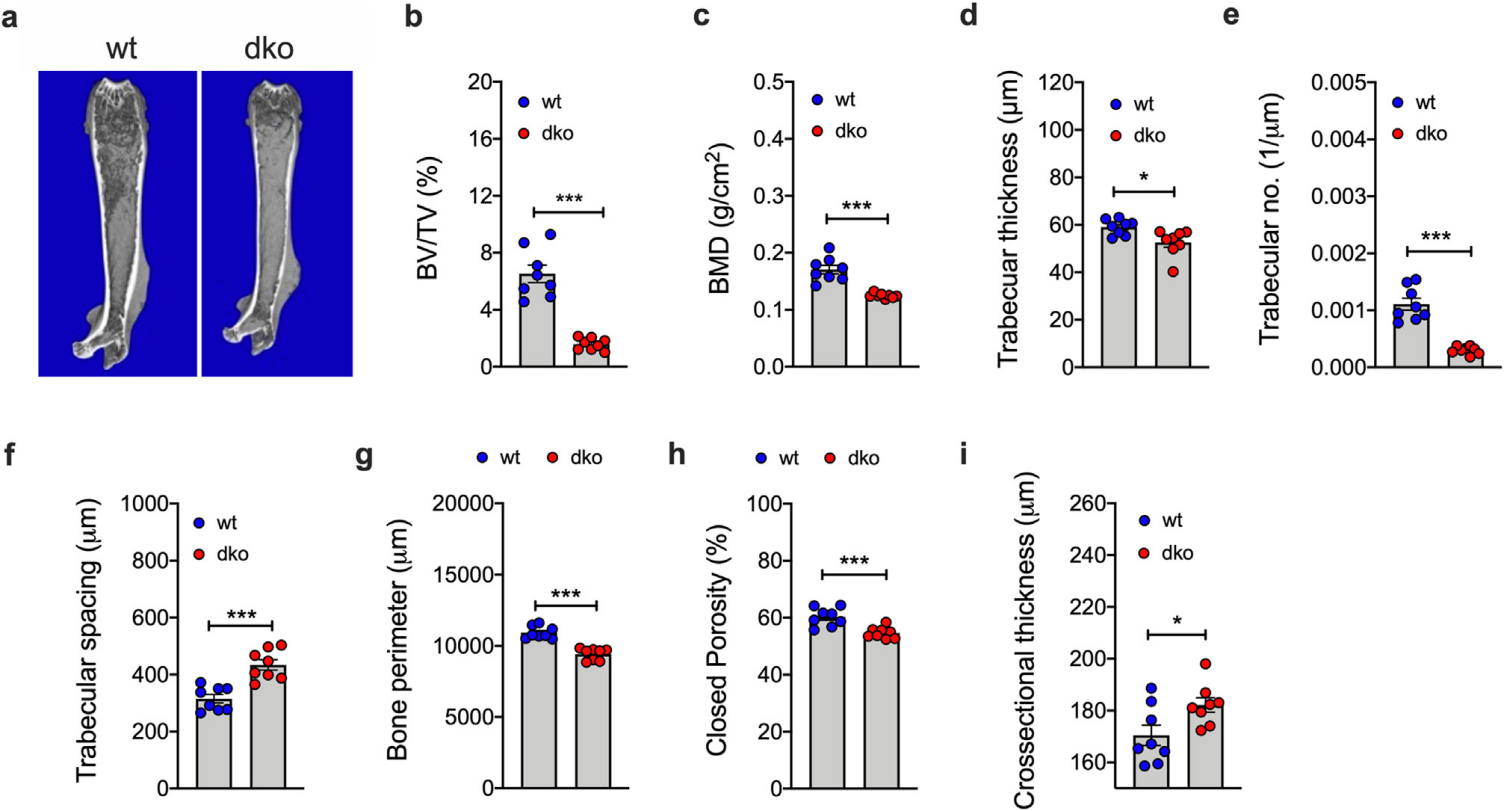
**Assessment of T3 effects on bone metabolism.** Representative MicroCT picture of the distal femur bone **(a)** and quantification of trabecular bone volume (BV) to tissue volume (TV) **(b)**, as well as bone mineral density (BMD) of trabecular bone **(c)**, trabecular thickness **(d)**, trabecular number **(e)**, trabecular spacing **(f)**, crossectional cortical bone perimeter **(g)**, closed porosity of cortical bone **(h)** and crossectional cortical bone thickness **(i)** in 20-wk old male *Mct8/Oatp1c1* dko mice and wildtype controls (n = 8 each group). Data were analyzed using student's two-tailed, two-sided ttest. Date represent mean ± SEM; asterisks indicate ∗ p < 0.05; ∗∗p < 0.01 and ∗∗∗p < 0.001.

Consistent with enhanced T3 action in the liver, muscle and adipose tissue, MicroCT analysis of the distal femur bone (Figure 4A) revealed a strong decrease in trabecular bone volume (Figure 4B) and bone mineral density (BMD) in *Mct8/Oatp1c1* dKO mice relative to wildtype controls (Figure 4C). Likewise, dKO mice display a decreased trabecular thickness (Figure 4D), reduced trabecular number (Figure 4E) and increased trabecular spacing (Figure 4F). A reduction is also seen in cortical bone parameters the mean total cross-sectional bone perimeter (Figure 4G) and in closed porosity (Figure 4H), while cross-sectional cortical bone thickness is increased (Figure 4I). Collectively, these data indicate that the peripheral hyperthyroidism observed in *Mct8/Oatp1c1* dKO mice is accompanied by enhanced T3 action in liver, adipose tissue and the bone.

### 3.3 Mct8/Oatp1c1 dKO mice show severe neuronal hypomyelination

**Figure 5:**
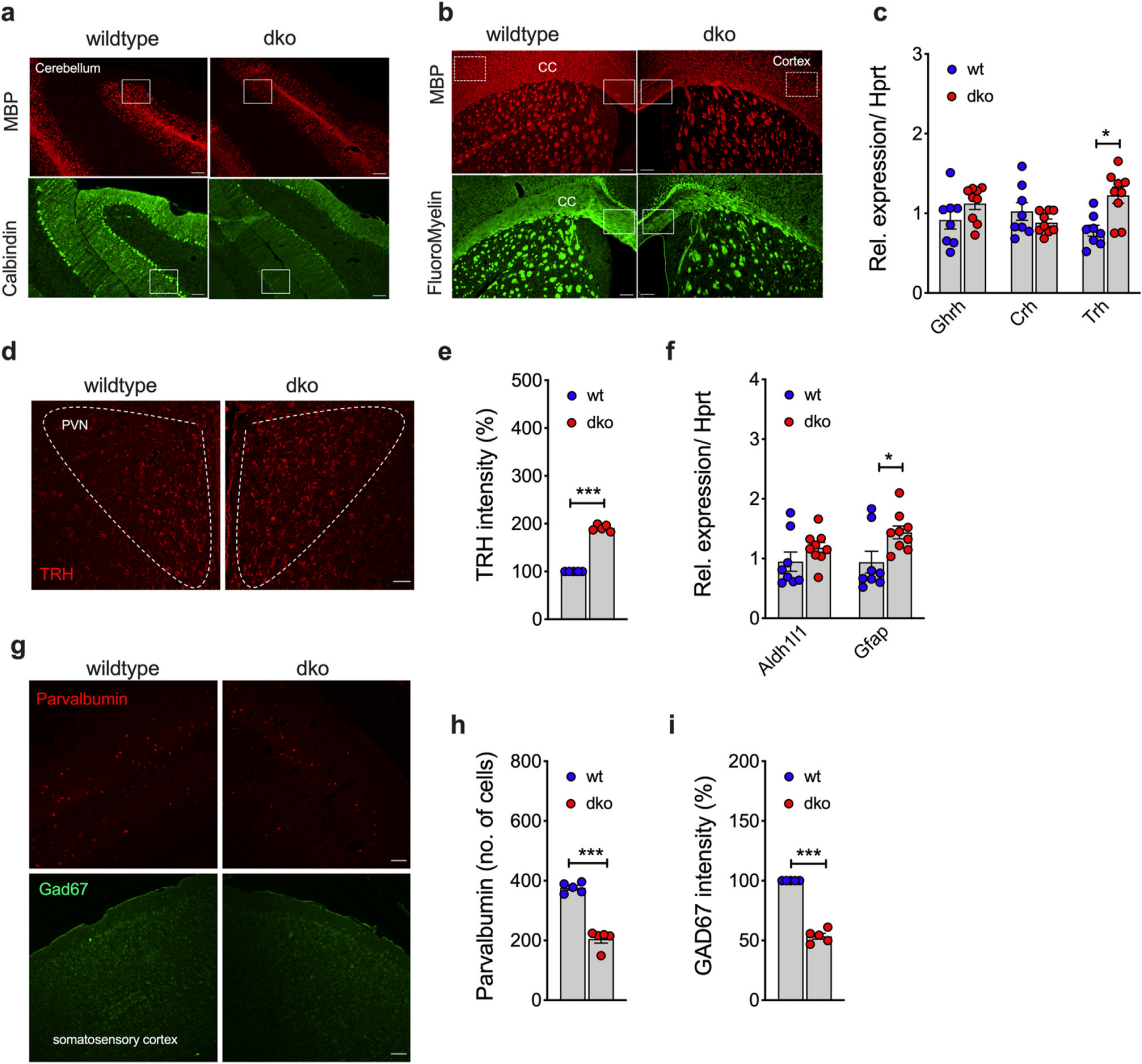
**Immunhistochemical analysis of neuronal myelination and mRNA quantification of hypothalamic target genes.** Representative immunofluorescent analysis of myelin expression using antibody against myelin basic protein (MBP) within the granular layer of the cerebellum and calbindin immunoreactivity in the Purkinje cell layer of chow-fed 24-wk old male mice (n = 5–6 per group; scale bar 100 μm) **(a)**. Representative fluorescent micrograph of total white matter distribution throughout the cerebral cortex, corpus callosum and basal ganglia depicted by Fluoromyelin staining and MBP immunoreactivity in 24-wk old chow-fed male mice (n = 5–6 per group; scale bar 100 μm) **(b)**. Differential expression of hypothalamic releasing hormones in 20-wk old chow-fed male mice (n = 8–9 each group **(c)**. Representative immunhistochemical staining **(d)** and quantification **(e)** of TRH in the hypothalamus of 24-wk old male mice (N = 5 each genotype; scale bar 100 μm). Expression of astrocytic marker genes *Aldh1l1* and *Gfap* in 20-wk old chow-fed male mice (n = 8–9 each group) **(f)**. Representative immunhistochemical staining **(g)** and quantification **(h,i)** of parvalbumin and Gad67 in the somatosensory cortex of 24 wk-old male mice (N = 5 each genotype; scale bar 100 μm). White squares in **(a**) and **(b)** show representative areas with neurological impairments. Data were analyzed using student's two-tailed, two-sided ttest. Data represent mean ± SEM; asterisks indicate ∗ p < 0.05; ∗∗p < 0.01 and ∗∗∗p < 0.001.

Deficiency of white matter due to hypomyelination of CNS neurons is commonly observed in patients with AHDS [15]. Consistent with this, we see cerebellar white matter markedly reduced in *Mct8/Oatp1c1* dKO mice, as indicated by immunhistochemical analyses of myelin basic protein (MBP) (Figure 5A). Impaired myelination in the granular layer is accompanied by degeneration of Purkinje fibers, as indicated by decreased immunostaining of calbindin within the adjacent molecular layer of the cerebellum (Figure 5A). Ex vivo Brain 10.13039/100011612MRI revealed a slight increase of T2 signal of the white matter in dKO mice relative to wildtype controls, further supporting hypomyelination in dKO mice (Suppl. Fig. 2F). *Mct8/Oatp1c1* dKO mice further show hypomyelination throughout the cerebral cortex and corpus callosum, as evidenced by Fluoromyelin and MBP immunostaining (Figure 5B). In the hypothalamus, we find expression of thyrotropin releasing hormone (*Trh*) increased in *Mct8/Oatp1c1* dKO mice (Figure 5C), and this is verified also using immunhistochemical analysis (Figure 5D,E). No changes are observed in expression of corticotropin releasing hormone (*Crh*) or growth hormone releasing hormone (*Ghrh*) (Figure 5C). We also see increased expression of the astrocyte-specific glial fibrillary acidic protein (*Gfap*) in *Mct8/Oatp1c1* dKO mice relative to wildtype controls, without alterations in aldehyde dehydrogenase 1 family member L1 (*Aldh1l1*) (Figure 5F), indicating that the decreased myelination coincides with enhanced reactive astrogliosis in the hypothalamus. Immunhistochemical analysis also revealed decreased abundance of parvalbumin and Gad67 in the somatosensory cortex (Figure 5G–I), suggesting that dKO mice show impaired cognitive behavior.

### 3.4 Mct8/Oatp1c1 dKO mice show impaired motor abilities

**Figure 6:**
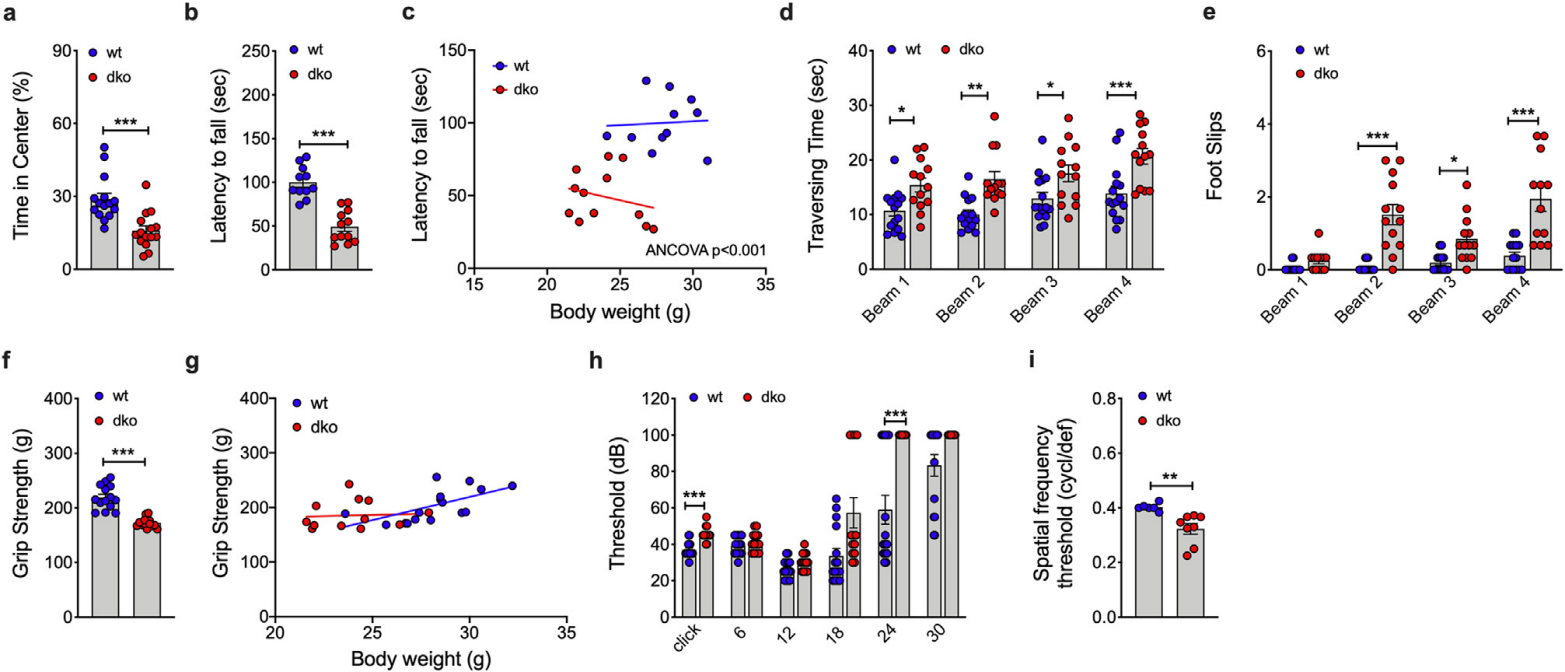
**Assessment of anxiety and motor ability in *Mct8/Oatp1c1* dKO mice.** Quantification of time spent in the center using the open field test in 9-wk old male *Mct8/Oatp1c1* dKO mice and wildtype controls (n = 15 each group) **(a)**. Latency to fall assessed on the rotarod in 13-wk old male mice (n = 11–12 each group) **(b, c)**, traversing time and foot slips assessed in the beam balance test in 19-wk old male mice (n = 12–15 each group) **(d,e)** and grip strengths in 13-wk old male mice (n = 13–15 each group) **(f,g)**. Stimulus intensity threshold measured in the auditory brainstem response (ABR) for different frequencies (kHz) **(h)** and spatial frequency threshold in the virtual drum in 24-wk old male mice (n = 13–15 and n = 6–8 each group respectively) **(i)**. Data in panel **a,b, d-f, h and i** were analyzed using student's two-tailed, two-sided ttest. Data in panel **c and g** was analyzed using ANCOVA with body weight as covariate. Date represent mean ± SEM; asterisks indicate ∗ p < 0.05; ∗∗p < 0.01 and ∗∗∗p < 0.001.

We next assessed whether the observed neurological alterations in *Mct8/Oatp1c1* dKO mice (Figure 5A,B) translate to behavioral and/or motor impairments. Unlike patients with AHDS, we see an increased anxiety-like behavior in *Mct8/Oatp1c1* dKO mice relative to wildtype controls, as indicated by reduced time spend in the center in the open field test (Figure 6A). On the accelerating rotarod, *Mct8/Oatp1c1* dKO mice display a decreased latency to fall, indicating that the dKO mice have, similar to patients with AHDS, impaired ability in motor learning and locomotor coordination (Figure 6B,C). Consistent with this, *Mct8/Oatp1c1* dKO mice show longer traversing time and an increased number of foot slips in the balance beam test (Figure 6D,E). Muscle grip strength is likewise reduced in *Mct8/Oatp1c1* dKO mice (Figure 6F) but is normal when considering the decreased body weight as covariate (Figure 6G). Collectively, these data indicate that *Mct8/Oatp1c1* dKO mice display, similar to patients with AHDS, severe motor impairments. The motor impairments are likely associated with the observed neurological alterations (Figure 5A,B) and are not mediated by alterations in muscle strenghts. The dKO mice further show a heightened threshold (Figure 6H) in auditory brainstem response (ABR), accompanied by a decreased spatial frequency threshold in visual testing (Figure 6I) relative to wt controls, collectively suggesting that *Mct8/Oatp1c1* dKO mice show impaired peripheral hearing and optomotor abilities.

## 4 Discussion

Key hallmarks of the AHDS are decreased thyroid hormone levels in the brain with elevated (T3) or decreased level (T4) in the periphery. These endocrine abnormalities manifest in severe cognitive and motor impairments, hypotonia, muscle hypoplasia, spasticity and severe intellectual disability [27]. Consistent with a chronic state of cerebral hypothyroidism and peripheral thyrotoxicity, patients with AHDS show increased mortality and reduced life expectancy [27]. While to this date there is no curative therapy, the development of pharmacotherapies to treat AHDS depends on the availability of a reliable model organism to assess treatment outcome. The main purpose of this study was to assess whether mice with concomitant deletion of *Mct8* and *Oatp1c1* may serve as such a model organism. We report the generation and metabolic characterization of a newly CRISPR/Cas9-generated global *Mct8/Oatp1c1* dKO mouse, and demonstrate that this mouse mimics a variety of key hallmarks seen in patients with AHDS. Similar to patients with AHDS, and consistent with previous reports [17], *Mct8*/*Oatp1c1* dKO mice show strikingly reduced levels of thyroid hormone T3 and T4 in the brain, with elevated levels of T3 and decreased levels of T4 in the periphery. The central hypothyroidism in *Mct8*/*Oatp1c1* dKO mice manifests in severe neuronal hypomyelination and motor impairments, as assessed by rotarod and the beam balance test. In line with the peripheral thyrotoxicity that is seen in patients with AHDS, *Mct8*/*Oatp1c1* dKO mice not only show strikingly enhanced levels of T3 in the circulation, but also in all peripheral tissues analyzed, namely the heart, liver, adipose tissue and the muscle. Enhanced peripheral T3 action is thereby supported in a variety of measures. Consistent with previous data [28], and the ability of T3 to enhance lipid metabolism, we see body weight and body fat mass decreased in *Mct8*/*Oatp1c1* dKO mice, and this is paralleled by decreased lipid size and reduced levels of plasma triglycerides. In line with the ability of T3 to enhance muscle and bone catabolism, we see decreased lean tissue mass and enhanced bone catabolism in *Mct8*/*Oatp1c1* dKO mice, while decreased circulating levels of cholesterol indicate enhanced T3 action in the liver. The enhanced bone catabolism is in agreement with recent reports in Mct8/Oatp1c1 dKO mice [29]. The observation that food intake is decreased in central hypothyroid dKO mice is likewise consistent with previous data [28] and underlines that T3 regulation of food intake is centrally mediated [12]. Also, the observation that dKO mice show normal locomotor activity and no signs of heart hypertrophy is consistent with previous reports [28]. But in contrast to previous data [28], we observed no indications of elevated BAT activity in dKO mice, and this is supported also by the demonstration that energy expenditure is not different between dKO mice and wildtype controls. Similar to patients with AHDS, we find severe neuroendocrine alterations in dKO mice, as well as increased mortality and reduced life expectancy. We hence conclude that *Mct8*/*Oatp1c1* dKO mice represent a robust and reliable model organism for the preclinical assessment of drugs designed to treat the AHDS. Underlining the physiological relevance of this model organism for the development of drugs to treat AHDS, viral-mediated central restoration of *Mct8* expression increased central T3 levels and improves motor function in *Mct8*/*Oatp1c1* dKO mice [30,31].

## Author contributions

GM, LS, AM, LB, LG, OVA, SMH, AH, CB, VIS and KB designed and performed experiments and analyzed and interpreted data. AO, CB, VIS and KB performed the micro CT and MRI analysis. MdA and SL analyzed samples. JT, AF, SMH, HF, VGD, WW and MHdeA supervised studies and interpreted data. CGC, HB, HK, PTP, PAM, RdiM and MHT participated in data interpretation, evaluation of data and editing of the manuscript. TDM conceptualized the project, supervised experiments, analyzed and interpreted data and wrote the manuscript together with GM.

## Data Availability

Data will be made available on request.
